# Complete Pathologic Response of Squamous Cell Carcinoma Abutting an Internal Mammary Bypass Graft

**DOI:** 10.1016/j.atssr.2022.12.011

**Published:** 2023-01-04

**Authors:** Adam Lam, Nicholas P. Campbell, Hyde M. Russell, Seth B. Krantz

**Affiliations:** 1 Department of Surgery, University of Chicago, Chicago, Illinois; 2 Department of Medicine, NorthShore University HealthSystem, Evanston, Illinois; 3 Department of Surgery, NorthShore University HealthSystem, Evanston, Illinois

## Abstract

We present a case of an advanced squamous cell carcinoma encroaching on a patient’s left internal mammary artery bypass graft. Tumor board consensus was to proceed with 2 cycles of neoadjuvant chemotherapy followed by resection. Intraoperatively, the left internal mammary artery bypass could not be safely dissected from the adjacent pleura, but frozen sections were negative for malignant transformation. Final pathologic examination showed a complete pathologic response to neoadjuvant chemotherapy, and surveillance imaging is now negative for recurrence 5 years postoperatively. Although malignant neoplasms invading into adjacent vasculature can post technical challenges during an operation, neoadjuvant therapy can downstage these tumors and make resection feasible without added morbidity.

Lung squamous cell carcinoma (SCC) can pose challenges in treatment, given its typically more advanced stage at diagnosis and characteristically more central location. SCC is associated with an up to 30% shorter median survival compared with other non–small cell lung cancers. Administration of neoadjuvant therapy, most commonly chemotherapy combined with radiation therapy, can improve the resectability of locally advanced tumors, especially with invasion into critical surrounding structures. We present a case of an advanced left upper lobe SCC impinging on a patient’s left internal mammary artery (LIMA) bypass graft that was successfully treated with neoadjuvant chemotherapy followed by resection while sparing the LIMA bypass.

A 76-year-old former smoker (60 pack-years) with a history of 3-vessel coronary artery bypass grafting (CABG) presented to our clinic with multiple weeks of persistent cough, hemoptysis, and weight loss. Clinical examination was unremarkable. Chest computed tomography (CT), however, revealed a left upper lobe mass and borderline enlarged mediastinal and hilar lymph nodes. Positron emission tomography scan showed hypermetabolism of the mass, left hilar lymph nodes, and a distal left paratracheal node. Endobronchial ultrasound with transbronchial biopsy of the mass confirmed a diagnosis of SCC but was nondiagnostic for the hilar and subcarinal lymph nodes. Mediastinoscopy and sampling of levels 2R/L, 4R/L, and 7 lymph nodes were negative for metastatic disease. Thus, the tumor was clinically staged as IIIA (T3 N1).

Operative planning was complicated by the fact that the mass abutted a patent LIMA bypass graft. Coronary CT angiography showed proximal compression of the artery from the surrounding neoplasm but patent flow in the distal bypass (Figure). Although chemotherapy combined with radiation therapy would have provided the best response rate in the neoadjuvant setting, tumor board consensus was to proceed with induction chemotherapy alone, given the risks of irradiating the adjacent LIMA bypass. He underwent 2 cycles of carboplatin and nab-paclitaxel with minimal adverse effects. The tumor demonstrated an excellent clinical response with a reduction in size from 9.1 × 5.5 cm to 6.0 × 3.2 cm and a change from a complete solid lesion to a primarily cystic cavity.

**Figure:**
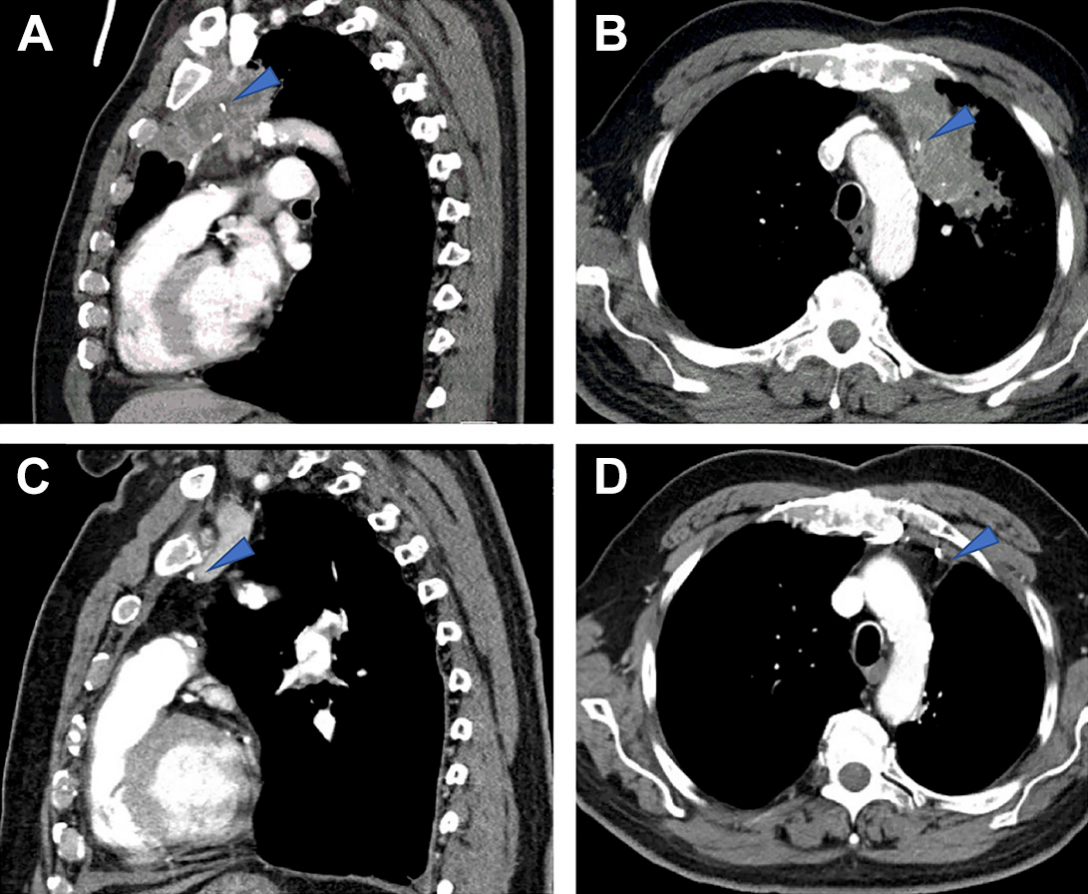
Pretreatment (A) sagittal and (B) axial views of left upper lobe squamous cell carcinoma abutting left internal mammary artery bypass. Postresection (C) sagittal and (D) axial views of the left side of the chest. Arrowheads point to left internal mammary artery.

In a joint operation involving both thoracic and cardiac surgeons, the patient underwent redo sternotomy with left upper lobectomy, regional lymphadenectomy, and total pulmonary decortication. Preoperative cardiac catheterization showed that the patient was completely dependent on the flow through the LIMA bypass. Vein mapping was performed, and both legs were prepared in case a venous graft needed to be obtained. Percutaneous access of the femoral artery and vein was also obtained. We were prepared to rapidly initiate cardiopulmonary bypass, which by offloading the heart would be protective against myocardial ischemia, although we were also prepared to cool the patient as needed for additional protection. During the dissection, the LIMA bypass was adherent to the pleura and could not be fully freed. Frozen sections of this pleural margin were negative for cancer, so the decision was made to leave behind a small pleural edge rather than to compromise the integrity of the bypass. The remainder of the postoperative hospital course was overall unremarkable, and the patient was discharged on postoperative day 5.

Final pathologic examination showed a complete pathologic response (ypT0 N0) with no residual primary tumor (0% viable tumor cells) and with 16 lymph nodes negative for metastatic disease. After resection, serial CT screenings have been negative for recurrence now up to 5 years postoperatively. He continues follow-up with the thoracic oncology service for serial surveillance.

## Comment

Lung cancer remains the leading cause of cancer-related death in the world. However, advances in neoadjuvant therapy are dynamically changing the landscape of lung cancer treatment. Platinum-based doublet chemotherapy like our patient received has traditionally been considered the standard of care for advanced lung SCC.^1^ However, rates of complete pathologic response are low for neoadjuvant chemotherapy. A review of neoadjuvant chemotherapy trials (including platinum-based doublet trials) for SCC found complete pathologic response rates between 0% and 16%.^2^ Our patient, who had a complete pathologic response to 2 cycles of nab-paclitaxel and carboplatin, was the exception, not the norm, for neoadjuvant doublet chemotherapy.

However, the exception may not be the case for long. Neoadjuvant immunotherapy has recently shown great promise for treating advanced lung cancer. Early trials assessing the addition of pembrolizumab or atezolizumab to neoadjuvant chemotherapy for advanced SCC have shown complete pathologic response rates of up to 45% to 50%.^3^^,^^4^ Immunotherapy in fact may soon replace radiation therapy in the neoadjuvant setting, especially because studies have suggested that the addition of radiation to induction chemotherapy does not improve survival^5^ and may be associated with worse outcomes.^6^

These innovations in neoadjuvant therapy have the potential to improve outcomes for all patients with lung cancer, improving resectability rates in patients with locally advanced cancers or in patients with other risk factors, such as a history of coronary bypass. A history of CABG can complicate lung resection by the presence of adhesive disease. In prior series, CABG patients undergoing lung resection were twice as likely to have postoperative hemorrhage (6.8% vs 3.5%; *P* = .009), and those with a LIMA bypass trended toward an increased rate of postoperative complications.^7^ Left upper lobe lesions can prove especially challenging, given their proximity to bypass grafts, but case series suggest that resection can be safely accomplished. A prior series of left upper lobe cancer patients with a history of LIMA bypass (n = 27) showed low morbidity and no short-term deaths after resection; however, two-thirds of these patients had early-stage cancers (16/27 stage 1 and 3/27 stage 2), and only 3 of 27 patients received neoadjuvant chemotherapy.^8^ In contrast, our patient’s tumor abutted the LIMA bypass, raising concern for the possible need for a redo bypass during resection. Fortunately, frozen specimens of this pleural border were negative for malignant transformation, allowing us to spare this pleural margin and to preserve the existing graft.

In conclusion, whereas SCC often is manifested with advanced disease, improvements in neoadjuvant therapy show promise in downstaging lesions and improving outcomes. Neoadjuvant therapy is crucial when lesions locally invade surrounding vasculature where resection poses high morbidity, and intraoperative biopsy should be strongly considered in these circumstances to avoid excessive dissection that may compromise these structures.
